# Prognostic factors of cervical node status in head and neck squamous cell carcinoma

**DOI:** 10.1186/s12957-015-0460-6

**Published:** 2015-02-15

**Authors:** Chairat Burusapat, Weerawut Jarungroongruangchai, Mongkon Charoenpitakchai

**Affiliations:** Division of Plastic and Reconstructive Surgery, Department of Surgery, Phramongkutklao Hospital and College of Medicine, 315 Ratchawithi Road, Thung Phayathai, Ratchathewi, Bangkok 10400 Thailand; Department of Pathology, Phramongkutklao College of Medicine, Bangkok, Thailand

**Keywords:** Head and neck squamous cell carcinoma, Cervical node metastasis, Prognostic factors

## Abstract

**Background:**

Cervical nodal status is one of prognostic factors in head and neck squamous cell carcinoma (HNSCC). The objective of this study was to identify prognostic factors of cervical node status including site and size of primary tumors, presence of lymphovascular invasion, and size of cervical node for appropriate further treatment in HNSCC.

**Methods:**

A 5-year retrospective review of patients with HNSCC in Phramongkutklao Hospital from 2009 to 2013 was conducted. Histopathologic data on primary tumors and cervical nodes were reviewed. Cervical nodes were divided into five groups: 1–3, 4–6, 7–9, 10–30, and >30 mm. Numbers of positive and negative nodes were compared in different sizes and sites and the presence of extracapsular extension.

**Results:**

In all, 165 patients and 1,472 nodes were reviewed. The mean age was 52.6 years and 77.58% were male. The most frequent primary site was oral tongue (50.91%). In sum, 52.72% showed lymphovascular invasion. Thirty-five patients (81.40%) in therapeutic neck dissections and 18 patients (69.23%) in prophylactic neck dissections showed nodal metastasis. The mean size of metastatic nodes was 3.89 mm (range, 2–45 mm) and 3.53 mm (range, 2–23 mm), respectively. Significant associations were found between the size of cervical nodes and the site of primary tumor of the oral tongue, lip, base of the tongue, and floor of the mouth (*p* < 0.05). Metastatic lymph nodes showed extracapsular extension 69.55%. No significance was found between extracapsular extension and clinical staging, size of primary tumor, pathologic differentiation, and size of cervical nodes. Sizes of cervical lymph node of squamous cell carcinoma (SCC) of the oral tongue and lip were statistically significant with the size of tumor and tumor grading (*p* < 0.05).

**Conclusions:**

A statistical significance was found between the size of cervical nodes and the site of primary tumor of the oral tongue and lip. Herein, we recommended performing neck dissection in all cases of SCC of the base of the tongue, floor of the mouth, buccal mucosa, and retromolar trigone.

## Background

Head and neck squamous cell carcinoma (HNSCC) is one of the most common malignant tumors of the skin and oral cavity. Early diagnosis and treatment have a favorable prognosis. Regional metastasis of HNSCC is most likely to involve the cervical lymph node. Nodal status is one of prognostic factor and affects the survival rate of patients. Then, accurate staging of cervical lymph node is necessary. Histopathologic confirmation of metastatic node is the method to provide the final staging.

The investigations including computed tomography (CT) [1,2], magnetic resonance imaging (MRI) [3-5], positron emission tomography (PET) [6,7], and Doppler ultrasound [8] give more information to identify lymph nodes. Moreover, the biomarker study for prognostic factors such as matrix metalloproteinase-13 (MMP-13) [9], cyclooxygenase-2 (COX-2) expression [10], and gene expression (molecular analysis) marker [11] can predict occult metastasis but the accuracy is inconclusive.

The size of cervical lymph node is the guideline for operative procedures. Usually, cervical nodes larger than 10 mm are significant for nodal metastasis and the operation is a radical neck dissection. However, pathologically identified neck node metastasis occurs in 34%–51% of prophylactic neck dissections and 58%–69% in therapeutic neck dissections [12,13].

At present, size criterion to diagnose occult malignant nodes is not reliable and presents a challenge for the surgeon to determine the extension of neck dissection. The association between nodal metastasis and size of cervical node is inconclusive and controversial.

The objective of this study was to identify prognostic factors of cervical node status including the site and size of primary tumors, presence of lymphovascular invasion, and size of cervical node for appropriate further treatment in HNSCC.

## Methods

Approval of this study was obtained from the ethics committee of Phramongkutklao Hospital and College of Medicine. A 5-year retrospective review of patients with HNSCC in Phramongkutklao Hospital from 2009 to 2013 was conducted.

Inclusion criteria were patients with squamous cell carcinoma (SCC) of the lips, oral tongue, base of the tongue, floor of the mouth, buccal mucosa, alveolar, and retromolar trigone. The patients with non-SCC and cancer of the thyroid, pharynx, larynx, tonsils, and salivary glands were excluded.

The database included age, sex, tumor site, tumor size, tumor staging, histopathologic data of primary tumors and cervical nodes, presence of cervical lymph node metastasis, and treatment modality. Histopathologic data of primary tumors included differentiation of tumor, presence of lymphovascular invasion, and perineural invasion. The differentiation of tumor was dependent on features such as nuclear and cytoplasmic differentiation and degree of keratinization and was divided in a four-grade system. Tumor differentiations were described as well differentiated, moderately differentiated, poorly differentiated, and undifferentiated.

The surgery of cervical neck nodes was divided into therapeutic neck dissection and prophylactic neck dissection. The therapeutic dissection was defined as neck dissection in patients with palpable cervical lymph nodes, or if the investigation showed cervical nodes larger than 8 mm, it was defined as radical neck dissection or modified neck dissection. Prophylactic neck dissection was performed in patients with tumor size more than 2 cm and all cases of the base of the tongue. Prophylactic neck dissection was defined as modified neck dissection or supraomohyoid neck dissection.

All cervical nodes were reviewed including the size of the node, presence of metastasis, and extracapsular extension under a microscope by an experienced pathologist. Cervical nodes were divided into five groups: 1–3, 4–6, 7–9, 10–30, and >30 mm. Cervical nodes were compared in different sizes, extracapsular extension, and number of positive and negative nodes at each site of HNSCC.

### Statistic analysis

Correlation between cervical nodes and tumors was analyzed. The univariate analysis of the independent variables was accomplished using Fisher’s exact test or chi-square test. A *p* value <0.05 was considered statistically significant.

## Results

A total of 204 patients with HNSCC were recorded. Thirty-nine patients were excluded because of refused treatment, comorbidity, distant metastasis, and unavailability of medical records. The remaining 165 patients with HNSCC were reviewed. Overall, the mean age of the patients was 52.6 years (range, 23 to 89 years) (Table 1). Of these patients, 50.3% ranged in age from 45 to 60 years. There were 128 (77.58%) male and 37 (22.42%) female. Oral tongue was the most frequent primary site (50.91%) and retromolar trigone was the least (3.03%). Clinical stage I, stage II, stage III, and stage IV were diagnosed in 60.00%, 13.94%, 13.94%, and 12.12%, respectively. Tumor sizes were classified in five categories, 22.42% were smaller than 1 cm and 39.39% ranged in sizes between 1.1 and 2.0 cm. Eighty-six patients (52.12%) were categorized as having well differentiated, 49 (29.70%) as moderately differentiated, 25 (15.15%) as poorly differentiated, and 5 (3.03%) as undifferentiated lesions.

**Table 1:** **Demographic data of 165 patients of HNSCC**

**Patient characteristics**	**Number**	**Percent**
Gender		
Male	128	77.58
Female	37	22.42
Age		
< 45 years	36	21.82
45–60 years	83	50.30
> 60 years	46	27.88
Site of SCC of the oral cavity		
Oral tongue	84	50.91
Base of the tongue	15	9.09
Lip	31	18.79
Floor of the mouth	19	11.51
Buccal mucosa	11	6.67
Retromolar trigone	5	3.03
Clinical staging		
Stage I	99	60.00
Stage II	23	13.94
Stage III	23	13.94
Stage IV	20	12.12
Tumor size		
≤ 1.0 cm	37	22.42
1.1–2.0 cm	65	39.39
2.1–3.0 cm	23	13.94
3.1–4.0 cm	27	16.36
> 4.0 cm	13	7.88
Pathologic differentiation		
Well differentiated	86	52.12
Moderately differentiated	49	29.70
Poorly differentiated	25	15.15
Undifferentiated	5	3.03

Sixty-nine patients (41.82%) underwent neck dissection and 1,472 nodes were found and reviewed (Table 2). The mean number of nodes was 21.33 nodes per patient. According to the histopathology reviewed, 87 patients (52.72%) showed lymphovascular invasion (Table 3). Lymphovascular invasion of SCC of the oral tongue, base of the tongue, lip, floor of the mouth, buccal mucosa, and retromolar trigone were 42.85%, 66.67%, 58.06%, 57.89%, 72.72%, and 80.00%, respectively. No statistical significance was found among lymphovascular invasion of primary tumor, clinical staging, and pathologic differentiation. A statistically significant association was found between the size of SCC of the oral tongue and lymphovascular invasion (*p* < 0.05) (Table 3). Thirty patients (69.77%) in therapeutic neck dissection and 15 patients (57.69%) in prophylactic neck dissection showed lymphovascular invasion of the primary tumor.

**Table 2:** **Characteristics of squamous cell carcinoma depend on the site of primary tumor**

	**Site of cancer**					
	**Oral tongue**	**Base of the tongue**	**Lip**	**Floor of the mouth**	**Buccal mucosa**	**Retromolar trigone**
	**84 (%)**	**15 (%)**	**31 (%)**	**19 (%)**	**11 (%)**	**5 (%)**
Clinical staging						
Stage I	68 (80.95)	3 (20.00)	19 (61.29)	4 (21.05)	5 (45.45)	0 (0.00)
Stage II	3 (3.57)	5 (33.33)	6 (19.35)	6 (31.58)	2 (18.18)	1 (20.00)
Stage III	8 (9.52)	3 (20.00)	3 (9.68)	5 (26.32)	2 (18.18)	2 (40.00)
Stage IV	5 (5.95)	4 (26.67)	3 (9.68)	4 (21.05)	2 (18.18)	2 (40.00)
Tumor size						
≤ 1.0 cm	22 (26.19)	1 (6.67)	11 (35.48)	2 (10.53)	1 (9.09)	0 (0.00)
1.1–2.0 cm	48 (57.14)	2 (13.33)	8 (25.81)	2 (10.53)	5 (45.45)	0 (0.00)
2.1–3.0 cm	4 (4.76)	4 (26.67)	7 (22.58)	6 (31.58)	2 (18.18)	0 (0.00)
3.1–4.0 cm	7 (8.33)	5 (33.33)	3 (9.68)	7 (22.58)	2 (18.18)	3 (60.00)
> 4.0 cm	3 (3.57)	3 (20.00)	2 (13.33)	2 (10.53)	1 (9.09)	2 (40.00)
Pathologic differentiation						
Well differentiated	50 (59.52)	6 (40.00)	15 (48.39)	8 (42.11)	7 (63.63)	0 (0.00)
Moderately differentiated	24 (28.57)	4 (26.67)	8 (25.81)	8 (42.11)	2 (18.18)	3 (60.00)
Poorly differentiated	7 (8.33)	4 (26.67)	7 (22.58)	3 (15.79)	2 (18.18)	2 (40.00)
Undifferentiated	3 (3.57)	1 (6.67)	1 (3.23)	0 (0.00)	0 (0.00)	0 (0.00)
Neck dissection						
Therapeutic	13 (15.48)	8 (53.33)	5 (16.13)	9 (47.37)	4 (36.36)	4 (80.00)
Prophylactic	3 (3.57)	7 (46.67)	7 (22.58)	6 (31.58)	2 (18.18)	1 (20.00)

**Table 3:** **Lymphovascular invasion of primary tumor**

	**Lymphovascular invasion**																	
	**Oral tongue**		***p*** **value** ^**a**^	**Base of the tongue**		***p*** **value** ^**a**^	**Lip**		***p*** **value** ^**a**^	**Floor of the mouth**		***p*** **value** ^**a**^	**Buccal mucosa**		***p*** **value** ^**a**^	**Retromolar trigone**		***p*** **value** ^**a**^
	**(84)**			**(15)**			**(31)**			**(19)**			**(11)**			**(5)**		
	**+**	**−**		**+**	**−**		**+**	**−**		**+**	**−**		**+**	**−**		**+**	**−**	
Clinical staging			0.127			0.825			0.595			0.898			0.547			NA
Stage I	25	43		2	1		12	7		2	2		2	3		0	0	
Stage II	2	1		2	2		2	4		4	2		1	1		1	0	
Stage III	6	2		2	1		2	1		3	2		2	0		2	0	
Stage IV	3	2		4	1		2	1		3	1		1	1		1	1	
Tumor size			0.012			0.800			0.266			0.546			0.662			NA
≤ 1.0 cm	3	19		0	1		6	5		0	1		1	0		0	0	
1.1–2.0 cm	23	25		1	1		7	1		1	1		3	2		0	0	
2.1–3.0 cm	3	1		3	1		2	4		4	2		2	1		0	0	
3.1–4.0 cm	5	2		4	1		2	1		4	3		3	0		2	1	
> 4.0 cm	2	1		2	1		1	2		2	0		1	1		2	0	
Pathologic differentiation			0.339			0.804			0.843			0.564			NA			NA
Well differentiated	22	28		3	3		9	5		6	2		5	2		0	0	
Moderately differentiated	8	16		2	2		4	5		4	4		2	0		2	0	
Poorly differentiated	5	2		1	3		4	3		1	2		1	1		0	0	
Undifferentiated	1	2		1	0		1	0		0	0		0	0		2	1	
Neck dissection			1.000			0.608			1.000			1.000			1.000			NA
Therapeutic	9	4		6	2		3	2		6	3		3	1		3	1	
Prophylactic	2	1		4	3		3	4		4	2		1	1		1	0	

^a^Chi-square test or Fisher’ s exact test.

+ primary tumor had lymphovascular invasion, − primary tumor did not have lymphovascular invasion.

Therapeutic neck dissections were performed in 43 patients (62.32%) and prophylactic neck dissections were performed in 26 patients (37.68%). Thirty-five patients (81.40%) in the therapeutic neck dissections and 18 patients (69.23%) in the prophylactic neck dissections showed metastasis of SCC to the cervical nodes. Forty-three patients underwent therapeutic neck dissection, producing 956 nodes (average 22.23 nodes per patient) and 26 patients in prophylactic neck dissection group generated 516 nodes (average 19.85 nodes per patient) (Table 4). The most common size of cervical nodes was 1–3 mm. The mean size of nodes was 3.67 mm (range, 1–45 mm). Metastatic nodes totaled 289 nodes (19.63%).

**Table 4:** **Size of neck node and metastasis**

**Size of neck node**	**Oral tongue**		***p*** **value** ^**a**^	**Base of the tongue**		***p*** **value** ^**a**^	**Lip**		***p*** **value** ^**a**^	**Floor of the mouth**		***p*** **value** ^**a**^	**Buccal mucosa**		***p*** **value** ^**a**^	**Retromolar trigone**		***p*** **value** ^**a**^
	**(410)**			**(319)**			**(238)**			**(231)**			**(159)**			**(115)**		
	**+**	**−**		**+**	**−**		**+**	**−**		**+**	**−**		**+**	**−**		**+**	**−**	
Therapeutic neck dissection			<0.001			0.072			0.012			0.002			0.231			0.682
1–3 mm	7	78		5	35		3	27		2	40		2	18		2	12	
4–6 mm	10	61		7	42		5	29		6	25		5	14		3	7	
7–9 mm	11	31		7	39		6	38		9	28		7	21		5	10	
10–30 mm	31	53		16	36		18	33		13	19		9	21		12	33	
> 30 mm	1	1		0	0		0	0		0	0		2	1		0	0	
Prophylactic neck dissection			0.006			0.013			0.150			0.007			0.628			0.898
1–3 mm	2	34		2	26		1	12		2	28		1	11		1	4	
4–6 mm	4	28		4	32		2	14		3	21		2	14		1	4	
7–9 mm	4	30		5	35		2	21		3	17		3	17		2	7	
10–30 mm	9	15		10	18		8	19		7	8		3	8		4	8	
> 30 mm	0	0		0	0		0	0		0	0		0	0		0	0	

^a^Chi-square test or Fisher’ s exact test.

+ metastasis of SCC to cervical lymph node, − no metastasis of SCC to cervical lymph node.

In all, 204 nodes (21.34%) in the group of therapeutic neck dissection showed metastasis, and the mean size of metastatic nodes was 3.89 mm (range, 2–45 mm). Eighty-five nodes (16.47%) in the prophylactic neck dissection group exhibited metastasis, and the mean size of metastatic nodes was 3.53 mm (range of metastasis nodes, 2–23 mm). In the therapeutic neck dissection group, a statistical significance was found between the size of cervical nodes and the site of primary tumor of the oral tongue, lip, and floor of the mouth (*p* < 0.05). In the prophylactic neck dissection group, a statistical significance was found between the size of cervical nodes and the site of primary tumor of the oral tongue, base of the tongue, and floor of the mouth (*p* < 0.05) (Table 4).

Among 289 metastatic nodes, 201 (69.55%) showed extracapsular extension (Table 5). No statistical significance was found among extracapsular extension of cervical nodes and clinical staging, size of primary tumor, pathologic differentiation, and size of cervical nodes. According to each site of tumor, the size of cervical lymph node of SCC of the oral tongue was statistically significant with size of tumor and tumor grading (*p* < 0.05) (Table 6). The size of cervical lymph node of SCC of the lip was statistically significant with clinical staging and tumor grading (*p* < 0.05) (Table 7). No statistical significance was observed among SCC of the base of the tongue, floor of the mouth, buccal mucosa, and retromolar trigone and clinical staging, size of primary tumor, and pathologic differentiation (Tables 8, 9, 10, and 11).

**Table 5:** **Analysis of extracapsular extension of metastasis cervical node in each site of SCC**

	**Extracapsular extension**																	
	**Oral tongue**		***p*** **value** ^**a**^	**Base of the tongue**		***p*** **value** ^**a**^	**Lip**		***p*** **value** ^**a**^	**Floor of the mouth**		***p*** **value** ^**a**^	**Buccal mucosa**		***p*** **value** ^**a**^	**Retromolar trigone**		***p*** **value** ^**a**^
	**(79)**			**(56)**			**(45)**			**(45)**			**(34)**			**(30)**		
	**+**	**−**		**+**	**−**		**+**	**−**		**+**	**−**		**+**	**−**		**+**	**−**	
Clinical staging			0.465			0.518			0.195			0.255			0.891			0.634
Stage I	0	0		5	5		0	0		0	0		0	0		0	0	
Stage II	6	4		11	6		9	8		8	6		3	2		2	2	
Stage III	25	11		9	5		12	3		12	3		10	4		9	5	
Stage IV	26	7		12	3		10	3		13	3		10	5		9	3	
Tumor size			0.121			0.318			0.433			0.865			0.898			1.000
≤ 1.0 cm	0	0		3	4		0	0		0	0		0	0		0	0	
1.1–2.0 cm	3	4		3	4		0	0		0	0		0	0		0	0	
2.1–3.0 cm	11	7		11	3		13	8		11	5		7	4		0	0	
3.1–4.0 cm	19	5		13	5		8	4		15	5		8	4		11	6	
> 4.0 cm	24	6		7	3		10	2		7	2		8	3		9	4	
Pathologic differentiation			0.874			0.447			0.718			0.474			0.654			0.705
Well differentiated	21	7		10	9		7	5		10	6		7	3		8	5	
Moderately differentiated	25	9		12	5		8	2		15	4		7	2		0	0	
Poorly differentiated	6	3		11	3		10	5		8	2		9	6		12	5	
Undifferentiated	5	3		4	2		6	2		0	0		0	0		0	0	
Size of neck node			0.506			0.793			0.849			0.968			0.824			0.994
1–3 mm	5	4		4	3		3	1		3	1		2	1		2	1	
4–6 mm	9	5		7	4		4	3		6	3		4	3		3	1	
7–9 mm	11	4		9	3		6	2		9	3		6	4		5	2	
10–30 mm	31	9		17	9		18	8		15	5		9	3		10	6	
> 30 mm	1	0		0	0		0	0		0	0		2	0		0	0	

^a^Chi-square test or Fisher’ s exact test.

+ extracapsular extension of metastasis cervical node, − no extracapsular extension of metastasis cervical node.

**Table 6:** **Size of cervical lymph node and metastasis in oral tongue squamous cell carcinoma**

	**Size of cervical node**														
	**1–3 mm**		***p*** **value** ^**a**^	**4–6 mm**		***p*** **value** ^**a**^	**7–9 mm**		***p*** **value** ^**a**^	**10–30 mm**		***p*** **value** ^**a**^	**>30 mm**		***p*** **value** ^**a**^
	**+**	**−**		**+**	**−**		**+**	**−**		**+**	**−**		**+**	**−**	
	**9**	**112**		**14**	**89**		**15**	**61**		**40**	**68**		**1**	**1**	
Clinical staging			0.354			0.794			0.576			0.320			NA
Stage I	0	0		0	0		0	0		0	0		0	0	
Stage II	0	9		2	8		4	11		4	12		0	0	
Stage III	3	54		6	44		5	29		22	28		0	0	
Stage IV	6	49		6	37		6	21		14	28		1	1	
Tumor size			0.033			0.467			0.915			0.140			NA
≤ 1.0 cm	0	0		0	0		0	0		0	0		0	0	
1.1–2.0 cm	1	61		3	10		3	9		0	0		0	0	
2.1–3.0 cm	3	15		4	18		6	24		5	15		0	0	
3.1–4.0 cm	2	24		3	36		3	17		16	33		0	0	
> 4.0 cm	3	12		4	25		3	11		19	20		1	1	
Pathologic differentiation			0.044			0.711			0.278			0.028			NA
Well differentiated	2	58		4	35		4	29		18	24		0	0	
Moderately differentiated	2	32		6	27		7	14		19	22		0	0	
Poorly differentiated	4	13		2	18		2	11		1	12		0	0	
Undifferentiated	1	9		2	9		2	7		2	10		1	1	

^a^Chi-square test or Fisher’ s exact test.

+ metastasis of SCC to cervical node, − no metastasis of SCC to cervical node.

**Table 7:** **Size of lymph node and metastasis in squamous cell carcinoma of lip**

	**Size of cervical node**														
	**1–3 mm**		***p*** **value** ^**a**^	**4–6 mm**		***p*** **value** ^**a**^	**7–9 mm**		***p*** **value** ^**a**^	**10–30 mm**		***p*** **value** ^**a**^	**>30 mm**		***p*** **value** ^**a**^
	**+**	**−**		**+**	**−**		**+**	**−**		**+**	**−**		**+**	**−**	
	**4**	**39**		**7**	**43**		**8**	**59**		**26**	**52**		**0**	**0**	
Clinical staging			0.344			0.492			0.238			0.015			NA
Stage I	0	0		0	0		0	0		0	0		0	0	
Stage II	0	14		1	16		1	17		15	15		0	0	
Stage III	2	12		3	14		2	23		8	16		0	0	
Stage IV	2	13		3	13		5	19		3	21		0	0	
Tumor size			0.804			0.832			0.832			0.081			NA
≤ 1.0 cm	0	0		0	0		0	0		0	0				
1.1–2.0 cm	0	0		0	0		0	0		0	0		0	0	
2.1–3.0 cm	2	14		3	17		3	18		13	15		0	0	
3.1–4.0 cm	1	16		2	17		2	21		7	19		0	0	
> 4.0 cm	1	9		2	9		3	20		6	24		0	0	
Pathologic differentiation			0.776			0.709			0.907			<0.001			NA
Well differentiated	1	14		2	18		3	18		6	27		0	0	
Moderately differentiated	2	11		2	14		2	18		4	19		0	0	
Poorly differentiated	1	10		2	9		2	11		10	4		0	0	
Undifferentiated	0	4		1	2		1	12		6	2		0	0	

^a^Chi-square test or Fisher’ s exact test.

+ metastasis of SCC to cervical node, − no metastasis of SCC to cervical node.

**Table 8:** **Size of lymph node and metastasis of squamous cell carcinoma of the base of the tongue**

	**Size of cervical node**														
	**1–3 mm**		***p*** **value** ^**a**^	**4–6 mm**		***p*** **value** ^**a**^	**7–9 mm**		***p*** **value** ^**a**^	**10–30 mm**		***p*** **value** ^**a**^	**>30 mm**		***p*** **value** ^**a**^
	**+**	**−**		**+**	**−**		**+**	**−**		**+**	**−**		**+**	**−**	
	**7**	**61**		**11**	**74**		**12**	**74**		**26**	**54**		**0**	**0**	
Clinical staging			0.280			0.976			0.602			0.999			NA
Stage I	1	11		2	11		3	9		4	8		0	0	
Stage II	1	23		3	25		4	28		9	19		0	0	
Stage III	2	18		3	19		2	21		7	14		0	0	
Stage IV	3	9		3	19		3	16		6	13		0	0	
Tumor size			0.441			0.413			0.756			0.161			NA
≤ 1.0 cm	0	8		1	5		1	7		5	6				
1.1–2.0 cm	1	14		2	5		2	5		2	6		0	0	
2.1–3.0 cm	1	17		2	27		2	21		9	12		0	0	
3.1–4.0 cm	3	15		3	27		4	21		8	13		0	0	
> 4.0 cm	2	7		3	10		3	20		2	17		0	0	
Pathologic differentiation			0.546			0.948			0.765			0.587			NA
Well differentiated	4	21		5	30		4	34		6	18		0	0	
Moderately differentiated	2	17		3	22		3	20		9	18		0	0	
Poorly differentiated	1	14		2	13		3	12		8	10		0	0	
Undifferentiated	0	9		1	9		2	8		3	8		0	0	

^a^Chi-square test or Fisher’ s exact test.

+ metastasis of SCC to cervical node, − no metastasis of SCC to cervical node.

**Table 9:** **Size of lymph node and metastasis of squamous cell carcinoma of the floor of the mouth**

	**Size of cervical node**														
	**1–3 mm**		***p*** **value** ^**a**^	**4–6 mm**		***p*** **value** ^**a**^	**7–9 mm**		***p*** **value** ^**a**^	**10–30 mm**		***p*** **value** ^**a**^	**>30 mm**		***p*** **value** ^**a**^
	**+**	**−**		**+**	**−**		**+**	**−**		**+**	**−**		**+**	**−**	
	**4**	**68**		**9**	**46**		**12**	**45**		**20**	**27**				
Clinical staging			0.591			0.598			0.437			0.946			NA
Stage I	0	0		0	0		0	0		0	0		0	0	
Stage II	1	23		2	16		5	13		6	7		0	0	
Stage III	1	27		3	17		5	16		6	9		0	0	
Stage IV	2	18		4	13		2	16		8	11		0	0	
Tumor size			0.859			0.913			0.347			0.871			NA
≤ 1.0 cm	0	0		0	0		0	0		0	0				
1.1–2.0 cm	0	0		0	0		0	0		0	0		0	0	
2.1–3.0 cm	1	19		3	17		5	18		7	8		0	0	
3.1–4.0 cm	1	24		4	17		6	15		9	12		0	0	
> 4.0 cm	2	25		2	12		1	12		4	7		0	0	
Pathologic differentiation			0.933			0.803			0.868			0.186			NA
Well differentiated	2	31		4	21		6	19		4	10		0	0	
Moderately differentiated	1	23		2	14		4	16		12	9		0	0	
Poorly differentiated	1	14		3	11		2	10		4	8		0	0	
Undifferentiated	0	0		0	0		0	0		0	0		0	0	

^a^Chi-square test or Fisher’ s exact test.

+ metastasis of SCC to cervical node, − no metastasis of SCC to cervical node.

**Table 10:** **Size of lymph node and metastasis in squamous cell carcinoma of buccal mucosa**

	**Size of cervical node**														
	**1–3 mm**		***p*** **value** ^**a**^	**4–6 mm**		***p*** **value** ^**a**^	**7–9 mm**		***p*** **value** ^**a**^	**10–30 mm**		***p*** **value** ^**a**^	**>30 mm**		***p*** **value** ^**a**^
	**+**	**−**		**+**	**−**		**+**	**−**		**+**	**−**		**+**	**−**	
	**3**	**29**		**7**	**28**		**10**	**38**		**12**	**29**		**2**	**1**	
Clinical staging			0.468			0.525			0.686			0.553			NA
Stage I	0	0		0	0		0	0		0	0		0	0	
Stage II	0	7		1	9		2	13		2	9		0	0	
Stage III	1	12		4	10		4	13		5	12		0	0	
Stage IV	2	10		2	9		4	12		5	8		2	1	
Tumor size			0.392			0.870			0.413			0.686			NA
≤ 1.0 cm	0	0		0	0		0	0		0	0		0	0	
1.1–2.0 cm	0	0		0	0		0	0		0	0		0	0	
2.1–3.0 cm	0	8		2	7		2	14		7	13		0	0	
3.1–4.0 cm	1	12		3	10		5	11		3	8		0	0	
> 4.0 cm	2	9		2	11		3	13		2	8		2	1	
Pathologic differentiation			0.988			0.975			0.603			0.240			NA
Well differentiated	1	11		3	12		2	14		4	12		0	0	
Moderately differentiated	1	9		2	9		4	12		2	10		0	0	
Poorly differentiated	1	9		2	7		4	12		6	7		2	1	
Undifferentiated	0	0		0	0		0	0		0	0		0	0	

^a^Chi-square test or Fisher’ s exact test.

+ metastasis of SCC to cervical node, − no metastasis of SCC to cervical node.

**Table 11:** **Size of lymph node and metastasis in squamous cell carcinoma of retromolar trigone**

	**Size of cervical node**														
	**1–3 mm**		***p*** **value** ^**a**^	**4–6 mm**		***p*** **value** ^**a**^	**7–9 mm**		***p*** **value** ^**a**^	**10–30 mm**		***p*** **value** ^**a**^	**>30 mm**		***p*** **value** ^**a**^
	**+**	**−**		**+**	**−**		**+**	**−**		**+**	**−**		**+**	**−**	
	**3**	**16**		**4**	**11**		**7**	**17**		**16**	**41**				
Clinical staging			0.484			0.880			0.931			0.231			NA
Stage I	0	0		0	0		0	0		0	0		0	0	
Stage II	0	5		1	3		2	5		1	11		0	0	
Stage III	1	5		1	4		3	6		9	18		0	0	
Stage IV	2	6		2	4		2	6		6	12		0	0	
Tumor size			0.582			1.000			0.659			0.082			NA
≤ 1.0 cm	0	0		0	0		0	0		0	0		0	0	
1.1–2.0 cm	0	0		0	0		0	0		0	0		0	0	
2.1–3.0 cm	0	0		0	0		0	0		0	0		0	0	
3.1–4.0 cm	1	9		2	6		3	10		11	17		0	0	
> 4.0 cm	2	7		2	5		4	7		5	24		0	0	
Pathologic differentiation			0.546			0.569			1.000			0.379			NA
Well differentiated	1	10		3	5		3	9		6	22		0	0	
Moderately differentiated	0	0		0	0		0	0		0	0		0	0	
Poorly differentiated	2	6		1	6		4	8		10	19		0	0	
Undifferentiated	0	0		0	0		0	0		0	0		0	0	

^a^Chi-square test or Fisher’ s exact test.

+ metastasis of SCC to cervical node, − no metastasis of SCC to cervical node.

Complications of neck dissection included wound infection, partial skin flap necrosis, and prolonged seroma. All complications were healed conservatively. Complications of neck dissection were found in six patients (13.95%) in therapeutic neck dissection and three patients (11.54%) in prophylactic neck dissection. No statistical significance was found among the complication rate between the therapeutic and prophylactic neck dissections.

## Discussion

HNSCC is one of the most common cancers. Morbidity and mortality are related to regional and distant metastasis. The number and size of cervical node metastasis varies depending on the site, tumor differentiation, and stage of primary tumor. Cervical node metastasis is an important prognostic factor for HNSCC [12]. Kuperman et al. revealed the relationship between the risk of distant metastasis and tumor site, size, and nodal status [14]. The size of the cervical lymph node remains an important factor in the interpretation of a clinically suspicious lymph node metastasis; however, it remains controversial regarding the significance of the size of the cervical node.

The accuracy of staging depends on the status of the cervical node. Many methods have attempted to detect the node status but no gold standard exists, except the histopathologic examination. Some studies have reported that the size of the cervical node was an inaccurate predictor of nodal metastasis and could not be regarded as an accurate means of staging in patients with clinically negative nodes [3,15]. Neck dissection is both a therapeutic and staging procedure and has evolved to include various types with standardized level designations (I–VI) for lymph node groups. Neck dissection is still a challenging treatment among patients with clinically negative nodes.

Ozer et al. suggested therapeutic neck dissection among patients with clinically negative nodes because pathologically positive nodes might be found in some patients [16]. Some reports have shown pathologically identified neck node metastasis occurred 34%–51% in prophylactic neck dissections [12,13]. In our study, 41.82% of HNSCC performed neck dissection. We found 69.23% of prophylactic neck dissections showed nodal metastasis with the size of the cervical lymph nodes varying from 2 to 23 mm. Statistically significant associations were found between the size of cervical nodes and the site of primary tumor of the oral tongue, base of the tongue, and floor of the mouth. No statistical significance was observed between the size of cervical nodes and the site of primary tumor at the buccal mucosa, lip, and retromolar trigone.

Di et al. reported the significance of the size of lymph node and recurrence. The size of the cervical node metastasis is the key risk factor in determining the development of cervical recurrence. Patients presenting extracapsular nodal spread and invasion of non-lymphatic structures have a high risk of developing cervical recurrence [17].

Fine needle aspiration (FNA) is one of the methods to identify cervical node metastasis, but negative FNA could not exclude metastasis at the cervical node. Sentinel node biopsy of the cervical node is controversial. Broglie et al. reported the occult metastases detected by sentinel node biopsy in patients with early HNSCC [18]. However, the current knowledge of sentinel lymph node biopsy does not allow avoiding the indication of elective neck dissection in clinical practice. Sentinel lymph node biopsy cannot be considered the standard of care at this time [19].

Many studies have attempted to identify preoperative cervical node status, but most of them have been usually inconclusive. Matsubara et al. indicated that F-18 FDG PET/CT was potentially useful in diagnosing preoperative nodal state [6]. Furthermore, a combined assessment of SUVmax with nodal size could be significant when identifying metastatic lymph nodes in HNSCC. Yamasaki et al. reported 80% false negative PET results in lymph nodes [7]. Kagawa et al. showed an increase in vascularity is a characteristic of Doppler ultrasound findings in small metastatic lymph nodes [8]. As the metastatic lymph node size increases, blood flow signals become scattered and the scattering index increases. Ding et al. demonstrated MRI diagnostic criteria of cervical lymph node metastasis include nodal size, central nodal necrosis, and irregular contour of lymph nodes [5]. Morphological MRI criteria have improved the detection of lymph node metastases in head and neck squamous cell carcinoma [4]. However, Feinmesser et al. argued that little advantage was achieved from MRI over clinical examination when detecting metastatic neck disease, and the present size criterion for the diagnosis of occult malignant nodes is not reliable [3].

Many methods have identified prognostic factors of HNSCC. Moreover, the biomarker study for prognostic factors such as MMP-13 [9], COX-2 expression [10], and gene expression (molecular analysis) marker [11] can predict occult metastasis, but the accuracy to detect occult metastasis is inconclusive.

Jan et al. reported the importance of tumor stage, surgical margin status, and extracapsular spread of cervical nodal metastasis as the most important prognostic factors in patients with buccal SCC [20]. Apisarnthanarax et al. found no significant difference between the extent of extracapsular extension and lymph node size. The incidence of extracapsular extension is associated with larger nodal size [21]. However, extracapsular extension is found in a substantial number of nodes with a diameter of <10 mm [22]. In our study, we found extracapsular extension 69.55% and no statistical significance among extracapsular extension of cervical nodes and staging, size of primary tumor, pathologic differentiated, and size of cervical node.

In our study, the size of cervical node ranges from 1 to 40 mm and the size of cervical node metastasis can be found as small as 2 mm. A statistical significance was found between the size of cervical nodes and the site of primary tumor of the oral tongue and lip (*p* < 0.05). We recommended performing neck dissection in all cases of SCC of the base of the tongue, floor of the mouth, buccal mucosa, and retromolar trigone. According to each site of tumor, the size of cervical lymph node of SCC of the oral tongue was statistically significant with the size of tumor and tumor grading. The size of cervical lymph node of SCC of the lip was statistically significant with clinical staging and tumor grading. However, the size of primary tumor and tumor grading association with the cervical nodes status were inconclusive.

In therapeutic neck dissection, statistically significant associations were found between the size of cervical lymph node and SCC of the oral tongue, lip, and floor of the mouth. In prophylactic neck dissection, statistically significant associations were observed between the size of cervical lymph node and SCC of the oral tongue, base of the tongue, and floor of the mouth. It meant that the larger the primary tumor, the higher the chance of metastasis, while SCC of the base of the tongue, floor of the mouth, buccal mucosa and retromolar trigone had the cervical node metastasis in small primary tumors. Herein, we recommended performing neck dissection, in cases of SCC of the base of the tongue, floor of the mouth, buccal mucosa, and retromolar trigone.

No statistically significant associations were found among extracapsular extension of the cervical node and clinical staging, size of primary tumor, pathologic differentiation, and size of cervical nodes. No statistically significant associations were observed among lymphovascular invasion of primary tumor, clinical staging, and pathologic differentiation. A statistically significant association was found between the size of SCC at the oral tongue and lymphovascular invasion. The importance of extracapsular extension and lymphovascular invasion is still unclear and further data is needed to support the prognosis.

One disadvantage of this study was that the pathologic status of the cervical node could not be determined in the patients which neck dissection was not performed. Additionally, we did not define the level of cervical nodes, despite the fact that cervical node levels I and II may increase the chance of node metastasis more than cervical node levels III and IV because this was a retrospective review with insufficient data. Moreover, a numerical difference between the tumor sites was observed, due to the incidence of sites of primary tumors was different. We did not study the survival rate of patients but emphasized the size of the metastatic cervical lymph node.

## Conclusions

The status of cervical lymph nodes is one of the prognostic factors in HNSCC. No gold standard exists, except the histopathologic examination to identify nodal status. The size of cervical node metastasis can be found as small as 2 mm. A statistical significance was found between the size of cervical nodes and the site of primary tumor of the oral tongue and lip. Herein, we recommended performing neck dissection in all cases of SCC of the base of the tongue, floor of the mouth, buccal mucosa, and retromolar trigone.

## Abbreviations

HNSCC: Head and neck squamous cell carcinoma
CT: Computed tomography
MRI: Magnetic resonance imaging
PET: Positron emission tomography
FNA: Fine needle aspiration
